# DeepNAPSI multi-reader nail psoriasis prediction using deep learning

**DOI:** 10.1038/s41598-023-32440-8

**Published:** 2023-04-01

**Authors:** Lukas Folle, Pauline Fenzl, Filippo Fagni, Mareike Thies, Vincent Christlein, Christine Meder, David Simon, Ioanna Minopoulou, Michael Sticherling, Georg Schett, Andreas Maier, Arnd Kleyer

**Affiliations:** 1 Pattern Recognition Lab, Department of Computer Science, Friedrich-Alexander-Universität Erlangen-Nürnberg, Martensstraße 3, 91058 Erlangen, Germany; 2 Department of Internal Medicine 3, Rheumatology and Immunology, Friedrich-Alexander-Universität Erlangen-Nürnberg and Universitätsklinikum Erlangen, Ulmenweg 18, 91054 Erlangen, Germany; 3 Deutsches Zentrum Immuntherapie, Friedrich-Alexander-Universität Erlangen-Nürnberg and Universitätsklinikum Erlangen, Ulmenweg 18, 91054 Erlangen, Germany; 4 Department of Dermatology, Friedrich-Alexander-Universität Erlangen-Nürnberg and Universitätsklinikum Erlangen, Ulmenweg 18, 91054 Erlangen, Germany

**Keywords:** Rheumatic diseases, Biomedical engineering, Computer science

## Abstract

Nail psoriasis occurs in about every second psoriasis patient. Both, finger and toe nails can be affected and also severely destroyed. Furthermore, nail psoriasis is associated with a more severe course of the disease and the development of psoriatic arthritis. User independent quantification of nail psoriasis, however, is challenging due to the heterogeneous involvement of matrix and nail bed. For this purpose, the nail psoriasis severity index (NAPSI) has been developed. Experts grade pathological changes of each nail of the patient leading to a maximum score of 80 for all nails of the hands. Application in clinical practice, however, is not feasible due to the time-intensive manual grading process especially if more nails are involved. In this work we aimed to automatically quantify the modified NAPSI (mNAPSI) of patients using neuronal networks retrospectively. First, we performed photographs of the hands of patients with psoriasis, psoriatic arthritis, and rheumatoid arthritis. In a second step, we collected and annotated the mNAPSI scores of 1154 nail photos. Followingly, we extracted each nail automatically using an automatic key-point-detection system. The agreement among the three readers with a Cronbach’s alpha of 94% was very high. With the nail images individually available, we trained a transformer-based neural network (BEiT) to predict the mNAPSI score. The network reached a good performance with an area-under-receiver-operator-curve of 88% and an area-under precision-recall-curve (PR-AUC) of 63%. We could compare the results with the human annotations and achieved a very high positive Pearson correlation of 90% by aggregating the predictions of the network on the test set to the patient-level. Lastly, we provided open access to the whole system enabling the use of the mNAPSI in clinical practice.

## Introduction

Psoriasis does not only affect the skin but also the nails, the joints and the entheses^1,2^. Nail psoriasis is a condition that is associated with a more severe disease, loss of quality of life and progress to psoriatic arthritis (PsA)^3,4^. Thus, in order to adapt the therapy of patients adequately and in a timely manner, quantifying the current extent of nail psoriasis as well as its progress over time is highly desirable. However, nail disease can be particularly difficult to diagnose, to quantify, and to treat adequately. The challenge consists of the heterogeneity of the manifestation with nail pitting, oil spots, crumbling, up to the complete detachment of the nail.

To quantify psoriatic nail disease the nail psoriasis severity index (NAPSI) has been developed^5^. The score allows the assessment of the severity of nail psoriasis by quantifying the pathological changes in the nails of patients. For this, a single nail is divided into four quadrants. Nail bed and nail matrix are scored for the presence of the following pathologies: nail matrix pathologies such as pitting, leukonychia, red spots in the lunula, and nail plate crumbling, and nail bed pathologies such as onycholysis, splinter hemorrhages, oil drop discoloration, and nail bed hyperkeratosis. Depending on the extent of the pathological changes, a score between one (one quadrant) to four (all quadrants affected) is given for the nail matrix and nail bed separately. The resulting score for a single nail lies between zero and eight and between zero and 80 for all nails of the hand. A shortcoming of the NAPSI is the strict division into quadrants and the necessity to be obtained during the patient visit. These shortcomings have been targeted by the modified nail psoriasis severity index (mNAPSI)^6^. Contrary to the original NAPSI, the score allows the retrospective analysis of the nails using photographs. Additionally, the nail is not subdivided into quadrants. The score consists of two sets of changes in the nail, which are graded separately: pitting, onycholysis and oil-drop discoloration, and crumbling, as well as leukonychia, splinter hemorrhages, hyperkeratosis, and red spots in the lunula. For a single nail, this leads to a maximum score of 8, and for both hands to a maximum score of 80.

Neural networks are becoming increasingly popular for tasks, that are time-intensive and repetitive. By adequately optimizing the internal parameters of the networks and with an appropriate amount of data, they can learn arbitrary mappings from the input data to the output, e.g. classify CT scans for the presence of a disease^7^. Lately, neural networks also were applied to challenges in the rheumatology, such as image-based classification of rheumatic diseases via data from magnetic resonance imaging or computed tomography^8,9^.

Even though the NAPSI has even been used in clinical trials to quantify the treatment effect^10,11^, the score is not used in clinical practice. The greatest constraint of the NAPSI is the time necessary for the grading of a single patient^12^. Recently, works started investigating the automated analysis of the nails using deep learning^13,14^. Hsieh et al. developed a custom box, that holds a custom camera and microcontroller to acquire the images. In total, they enrolled 45 patients in their work. The neural network used to detect and classify the images was a Mask R-CNN^15^. Ji et al. used a photo camera to acquire the images of 875 subjects. The neural network used was the Faster R-CNN^16^. The prior work has shortcomings. First, using the NAPSI as a classification target is not ideal, since it is not developed for retrospective analysis as the modified NAPSI. Second, the quadrant separation poses another difficulty, since a further algorithm to subdivide each nail is necessary. Additionally, both works only report classification performance on the nail level and fail to put this into the perspective of the patient-wise prediction of the NAPSI. Lastly, the detection of the nails does not need to be implemented anew, since openly-accessible tools already exist^17^.

In this work, we take the shortcoming of recent work into account and propose our approach:

- Standardized capturing of hand photos using a custom camera setup and a newly developed mobile device app.
- Acquisition of 1154 images of patient’s nails and reading of each nail with three independent readers.
- Extraction of the nails from the hand photos and training of a classification network to predict the NAPSI.
- Access to the complete method online.
- Patient-level NAPSI prediction comparison with human annotations.

## Methods

### Data recording, preparation, and annotation

**Figure 1:**
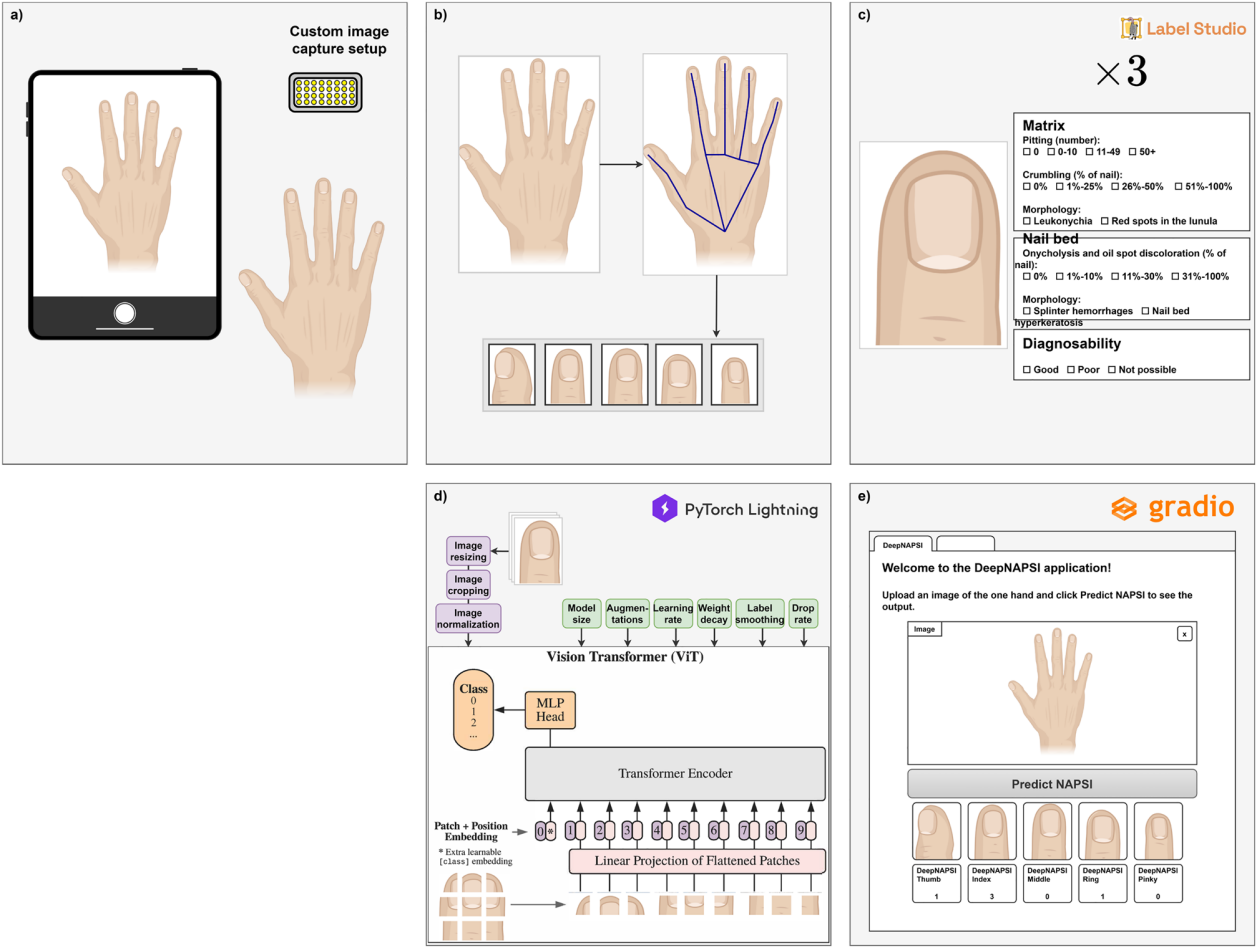
Overview of the proposed method. (**a**) The first step is the recording and storing of hand photos of patients using a custom setup and a purpose-built mobile application. (**b**) Based on key points extracted from the hand images using mediapipe, the location of each nail can be determined. (**c**) Each nail is graded by three independent readers according to the modified nail psoriasis severity index (mNAPSI). (**d**) A neural network (BEiT) is trained to classify the mNAPSI score of the presented nail. (**e**) Open-access to the trained system is provided upon request resulting in the network-predicted mNAPSI score for each hand.

Consecutive patients in the outpatient clinics of the Department of Internal Medicine 3, Rheumatology and the Department of Dermatology were approached to participate between January 2022 and November 2022 at the University Hospital Erlangen. Ethics approval to analyze the images was obtained from the ethics committee of Friedrich-Alexander-Universität (Dec. 2021/No. 21-422-B) and patients provided informed consent. Further, the disease of the patients were recorded. Images of each hand of the patients were acquired using a custom setup (Fig. 1a). A photo box with standardized lighting conditions using a photo light and the main camera of a mobile device (iPad Pro 2nd generation) was used. Further, a mobile application was developed to systematically store the acquired images and the corresponding patient id.

Only the nail region of the hand photos are relevant for the NAPSI classification. Hence, the hand key point detection algorithm of the mediapipe framework was used^17^. The algorithm computes the key points for each joint of the hands. Using the most distal key points of each finger the location of the nails could be extracted. Further, the orientation of the nail was corrected by rotation of the extracted nail at the angle determined using the most distal and second most distal key point of the finger. This results in a collection of ten normalized nail images for each patient as seen in Fig. 1b.

For the annotation of the modified NAPSI, all photos of the nails were graded by all three rheumatologists or rheumatologists in training (AK, PF, FF) with certified experience in the NAPSI according to the GRAPPA network training. Readers were blinded to the other nails and the disease and scored the nails retrospectively. Randomly, a single nail was presented to each reader without access to the grading of the other readers (Fig. 1c). The assessment according to the modified NAPSI (mNAPSI) consisted of three matrix and two nail bed assessments. Additionally, a diagnosability rating (good, poor, and no classification possible) was added for each nail to remove cases, where either the nail was not fully visible, hidden behind nail polish, or any other condition that hindered the mNAPSI assessment. A custom label studio interface was developed for the nail grading.

### mNAPSI classification network

For this work, a neural network was used that can predict a set of classes after being provided with an input image. Early experiments with state-of-the-art neural networks for natural image classification yielded a transformer-based network (BEiT^18^, 86M parameters) pre-trained on image patch prediction as the most promising method (Fig. 1d). A hyper-parameter search with validation area-under-receiver-operator-curve (AUROC) as target metric yielded the following best-performing configuration: Dropout rate of 6e-6, Adam optimizer with a learning rate of 9e-6, weight decay of $1e^{-3}$, and label smoothing of 3e-5. For the transformer network, a patch size of $16\times 16$ and an image size of $384\times 384$ was selected empirically. After suitable hyper-parameters were determined, the network was applied to predicted each case of the test set. Followingly, the patient-level performance of the neural network could be assessed.

### Pre-processing and augmentation

To prepare the nail photos for the training of the neural network, the images are processed beforehand. In addition, augmentation steps help the neural network become robust to the image variations common in our setup. Pre-processing steps were selected to match the pre-processing steps of the pre-training phase of the model and consisted of resizing the images to $384\times 384$ pixels and normalization of the mean and standard deviation of each sample to (0.485, 0.456, 0.406) and (0.229, 0.224, 0.225), respectively for each of the three RGB channels. The augmentations applied during the training of the networks consisted of random horizontal and vertical flips as well as random affine transformation with angles between -10 and +10 degrees, scaling between 90% and 110%, and translation between -10% and +10%. Each augmentation was applied with a probability of 50%. The obtained dataset was split into a training set (64%), a validation set (16%), and a test set (20%), taking care that all nail images of a patient are only present in either of the sets and the mNAPSI class distribution is preserved among all sets.

### DeepNAPSI access

To increase the availability of our approach, a custom, easy-to-use web-interface based on *Gradio*^19^ was developed. As seen in Fig. 1e, users can either upload a photo of the hand or directly capture a photo using any device with a camera. This image follows the same path described above, including key-point detection, nail extraction, and nail classification. Lastly, each extracted nail and the corresponding mNAPSI score as well as the mNAPSI score aggregate for the whole hand is provided to the users. Hosting of the model and the interface with GPU acceleration is provided by hugging face. Access to the system is available upon request. *Note*: This is not a medical product and cannot be used for the diagnostic purposes.

### Statistics

Inter-reader reliability of the mNAPSI scores aggregated to patient level was determined using Cronbach’s alpha^20^, similarly to Cassell et al.^6^. The correlation between reader ratings of the mNAPSI and the network-based predictions was determined using the Pearson correlation coefficient. In the test set, that was the basis of the comparison, only patients with complete annotations (all 10 nails) were present. Confidence intervals are reported at the confidence level of 95%.

## Results

### Dataset

After the removal of cases with any obstructions of the nail (N = 9), the collected dataset of patient photos consisted of 177 patients leading to 1154 nail photos. The mean age of patients was 53 years (SD 15 years) and slightly more female patients (56%, N = 99) than male patients (44%, N = 78) were collected. As the subjects were recruited consecutively at the clinic, a large amount were patients with a clinical diagnosis of rheumatoid arthritis (38%, N = 67), psoriatic arthritis (34%, N = 60), and psoriasis without signs of arthritis (10%, N = 18) were available. Additionally, 18% of the participants (N = 32) did not have a diagnosis of psoriasis or rheumatic disease at the time of recording.

**Figure 2:**
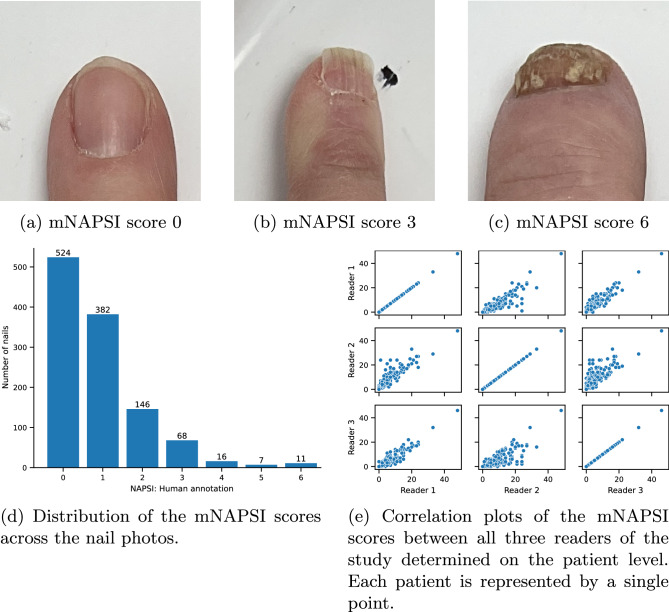
Overview of the acquired dataset including exemplary nail photos and corresponding mNAPSI grades, the distribution of the mNAPSI grades, and the agreement of all readers.

The reading of the nail photos by three experts yielded a skewed distribution of mNAPSI grades among all nails as seen in Fig. 2d. Most nails were assigned low mNAPSI scores: 524 nails with mNAPSI 0 and 382 with mNAPSI 1. Substantially fewer cases were available for higher mNAPSI scores: 146 for mNAPSI 2, 68 for mNAPSI 3, 16 for mNAPSI 4, 7 for mNAPSI 5, and 11 for mNAPSI 6. The highest scores for a single nail, mNAPSI 7 and 8, were not present in the dataset. As the training of a neural network requires a fundamental amount of samples across all targets, cases with mNAPSI scores higher or equal to 4 were aggregated in class 4 leading to 34 samples in the class. Exemplary samples of different mNAPSI scores are shown in Fig. 2a–c.

The agreement of the assigned mNAPSI scores across all three readers on a patient level is visualized in Fig. 2e. High inter-reader reliability of 0.94, 95% CI [0.92 0.95] was achieved, which is also in accordance with previous work^6^. A Pearson correlation between reader 1 and reader 2 of 0.86, CI [0.81, 0.89], between reader 1 and reader 3 of 0.93, CI [0.90, 0.94], and between reader 2 and reader 3 of 0.77, CI [0.70, 0.82] was observed. All p-values were smaller than $1e^{-4}$.

Patients without a diagnosis of psoriasis and PsA had a mNAPSI score greater than 0 for 91% of the patients (47% of the photos). Further, of all photos, 71% were assigned with a good diagnosability, 22% with a poor diagnosability, and 7% with no diagnosis possible.

### Classifier performance

The neural network to predict the mNAPSI scores for individual nails reached a good performance as seen in Table 1. Averaging the performance on each sample leads to the micro average results: AUROC 0.88, mean-average-error 0.5, F1 score 0.55, area-under-precision-recall curve 0.63, sensitivity 0.55, specificity 0.89, and accuracy 0.55.

**Table 1:** Class-specific and average performance metrics for the nail-wise mNAPSI classifier.

	AUROC	MAE	F1	PR-AUC	Sensitivity	Specificity	Accuracy
Class 0	0.86		0.70	0.83	0.62	0.88	0.62
Class 1	0.69		0.56	0.48	0.71	0.58	0.71
Class 2	0.80		0.24	0.35	0.22	0.92	0.22
Class 3	0.85		0.09	0.18	0.07	0.96	0.07
Class 4	1.00		0.00	0.98	0.00	1.00	0.00
Average (macro)	0.84	0.50	0.32	0.56	0.33	0.87	0.33
Average (micro)	0.88		0.55	0.63	0.55	0.89	0.55

Macro average is performed by averaging the class-wise metrics with equal weight, while the micro average is performed by averaging the class-wise metrics with weights proportional to the number of samples from the specific class. Classes 0 to 3 represent the corresponding mNAPSI scores for a single nail. Class 4 is an aggregate of all higher mNAPSI scores.

**Figure 3:**
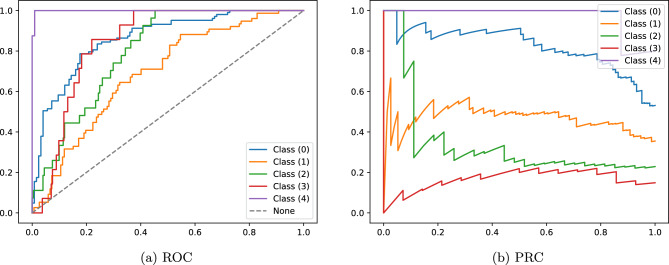
Receiver-operator-curve (ROC) and precision-recall-curve (PRC) of the classifier for each of the five aggregated mNAPSI classes.

The corresponding class-wise receiver-operator curves (ROC) and precision-recall curves (PRC) are depicted in Fig. 3a,b. The ROC and the PRC show very high performance of the classifier for samples of class 4 and class 0.

**Figure 4:**
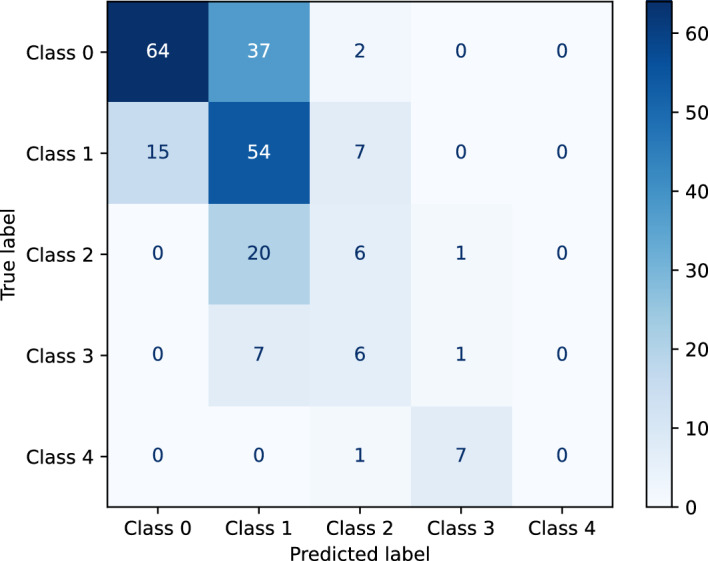
Confusion matrix of the ensemble of classification networks using the class with the highest probability in the prediction as the final prediction.

The confusion matrix in Fig. 4 provides a detailed insight into the correct and false predictions of the classification network. In general, the main weight of the matrix is distributed among the diagonal axis. For higher mNAPSI scores, the prediction deviates slightly from the diagonal axis, with samples from class 4 being predicted as class 2 and class 3. The inaccuracies for classes 3 and 4 are also reflected in the class-specific metrics.

### Patient-level mNAPSI performance

**Figure 5:**
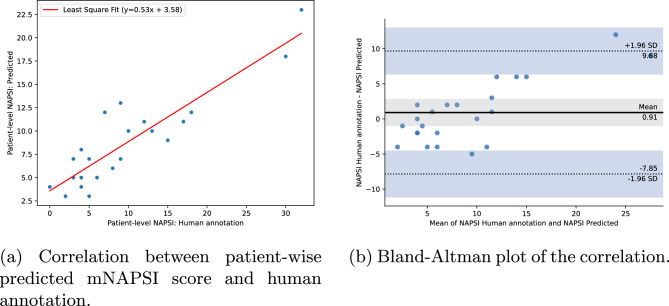
Correlation analysis of the predicted and human-annotated mNAPSI scores on the patient level.

As the final target of this work is the assessment of the complete mNAPSI score for a patient, the predictions of the network were aggregated to the patient level. For this, all classifier predictions were summed up for each patient. A correlation analysis between patient-wise mNAPSI prediction and human annotation depicted in Fig. 5 resulted in a very high positive Pearson correlation of 90.0% ($p < 1e^{-4}$). A least-squares fit to the points in Fig. 5a resulted in a slope of 0.53 and an intersection of 3.58. The Bland–Altman plot in Fig. 5b provides further insights into the correlation between network prediction and human annotation. The difference between both is relatively small with a mean difference of 0.91. Most cases are clustered close to the mean with some outliers at higher, relatively positive mNAPSI scores indicating a slight underestimation of the network.

## Discussion

In this work we were able to train and validate a neuronal network to predict the mNAPSI score of each finger automatically. Using our set up of a custom photo box, annotation tool, and neural network we were able to achieve remarkable results. In contrast to previous works, we did not only perform a classification of the nail images but also translated this to the patient-level performance and provide access to the complete system.

The proposed system is able to predict the mNAPSI for all nails of the hand at once. This enables not only the usage in clinical trials but also in the clinical practice. With increasing amounts of training data, the performance of the method will likely improve further. Further, the availability of three expert reader labels poses a very strong foundation for the neural network training. Environmental factors that might influence the ability of the method are the background of the hands during the recording and the lighting conditions.

Even though the results are motivating, there are some shortcomings that need to be addressed. First, the skin pigmentation of patients were mostly light with no cases of darker skin tones. Thus, the classification system might perform worse for patients with darker skin. We seek to improve our method in this regard in the future by selectively recruiting more pronounced pigmentations. Second, patients were only recruited at a single institution and a single device. We cannot rule out, that the application of our method on different devices might yield different results. Thus, we seek to record paired data using the existing setup and a further, different mobile device that is exposed to different lighting conditions in a less strict setup. Third, our dataset consisted of mostly low mNAPSI scores and grades greater or equal to mNAPSI 4 were grouped in class 4. Hence, the system can predict a patient score of at most 40. Thus, for patients with severe nail psoriasis, this will likely result in an underestimation of the mNAPSI score. In the future, we seek to recruit patients with higher mNAPSI scores. Fourth, the capturing setup relied on the flat positioning of all nails. For some patients, this was not possible due to the effects of the disease. Thus, we had to remove those patient photos from the dataset. In the future, we seek to optimize the capturing setup to also accommodate those cases by improving the ergonomics of our system. The correlation between nail psoriasis and psoriatic arthritis assumed in this work has a substantial impact on the prescription of medication for patients. Future work needs to investigate this relationship in detail in an unbiased manner. Lastly, the modified NAPSI score used in this work does not have an ideal correlation with clinical severity of nail psoriasis^21^. Thus, Baran’s severity index^22^ might be a more adequate choice for future work. Further, predicting the individual components of the severity index might also be beneficial, since this would allow a deeper understanding of the prediction of the neural network.

The contrast between high AUROC and PR-AUC and low accuracy for class 4 might seem counterintuitive. An explanation for this lies in the way in which the metrics are calculated. The accuracy is a threshold-based metric, which is sensitive to the selection of the exact threshold. On the other hand, the AUROC and PR-AUC are calculated by varying the threshold and measuring the effect on the prediction. This way the latter metrics are not sensitive to the threshold selected. Lowering the threshold for higher mNAPSI classes likely would improve the performance of those classes, however, at the same time, the performance of lower classes would presumably worsen. Thus, we decided to hold on to the initial choice of thresholds. The recruitment of patients with higher mNAPSI scores, as indicated above, would likely also improve the performance. The false predictions for classes 3 and 4 of the neural network seen in the confusion matrix are a limitation of the classifier. However, looking at the correlation plots of the reader, systematic difference in the grading process are also noted. The second reader gave consistently higher mNAPSI scores across all patients in comparison with both, reader 1 and reader 3. Thus, the final prediction of the mNAPSI score for a patient by the proposed system is likely in the range of the inter-reader reliability.

Translating the findings of this work to a system that can be used by patients at home requires further steps, since the environmental conditions will be vastly different from the controlled capturing setup used in this study. In the future, we seek to take this path by simultaneously acquiring photos using the existing setup and by asking the patients to record the photo themselves. Thereafter, the algorithm can be evaluated on the patient captured photos.

## Conclusion

In this work, we successfully recorded a dataset of patients and created nail psoriasis severity index (mNAPSI) annotations with the help of three experts. Followingly, we trained a neural network for the classification of the mNAPSI score on single nail images, which were extracted using key points of the hand photos. The classification network reached good performance with an AUROC of 88%. Next, we compared the human annotations and the network predictions aggregated to the patient level resulting in a very high positive Pearson correlation of 90%. Lastly, we provided access to the whole method upon request, enabling the use of the mNAPSI in clinical trials and clinical practice. In the future, we seek to deploy an app for patient use of our system at home. This has the potential to reduce patient visits to the clinic and, thus, reduce costs, while also enabling a more frequent assessment of the mNAPSI.

## Data Availability

The datasets used and analysed during this study are available from the corresponding author on reasonable request.
