# Author Correction: DeepNAPSI multi-reader nail psoriasis prediction using deep learning

**DOI:** 10.1038/s41598-023-37790-x

**Published:** 2023-06-30

**Authors:** Lukas Folle, Pauline Fenzl, Filippo Fagni, Mareike Thies, Vincent Christlein, Christine Meder, David Simon, Ioanna Minopoulou, Michael Sticherling, Georg Schett, Andreas Maier, Arnd Kleyer

**Affiliations:** 1 Pattern Recognition Lab, Department of Computer Science, Friedrich-Alexander-Universität Erlangen-Nürnberg, Martensstraße 3, 91058 Erlangen, Germany; 2 Department of Internal Medicine 3, Rheumatology and Immunology, Friedrich-Alexander-Universität Erlangen-Nürnberg and Universitätsklinikum Erlangen, Ulmenweg 18, 91054 Erlangen, Germany; 3 Deutsches Zentrum Immuntherapie, Friedrich-Alexander-Universität Erlangen-Nürnberg and Universitätsklinikum Erlangen, Ulmenweg 18, 91054 Erlangen, Germany; 4 Department of Dermatology, Friedrich-Alexander-Universität Erlangen-Nürnberg and Universitätsklinikum Erlangen, Ulmenweg 18, 91054 Erlangen, Germany

Correction to: *Scientific reports* 10.1038/s41598-023-32440-8, published online 01 April 2023

The original version of this Article contained an error in the spelling of the author Ioanna Minopoulou, which was incorrectly given as Ioanna Minnopoulou.

The original Article has been corrected.

